# A pilot study on feasibility and hypothesis exploration: reducing on-scene length of stay of the emergency teams via ambulance dispatch teleconsultation for prehospital examination

**DOI:** 10.31744/einstein_journal/2025AO1469

**Published:** 2025-04-25

**Authors:** Tarso Augusto Duenhas Accorsi, João Carlos Barbosa, Ricardo Galesso Cardoso, José Leão de Souza, Karine De Amicis, Renata Albaladejo Morbeck, José Paulo Ladeira, Eduardo Cordioli, Carlos Henrique Sartorato Pedrotti

**Affiliations:** 1 Telemedicine Department Hospital Israelita Albert Einstein São Paulo SP Brazil Telemedicine Department, Hospital Israelita Albert Einstein, São Paulo, SP, Brazil.; 2 Emergency Department Hospital Israelita Albert Einstein São Paulo SP Brazil Emergency Department, Hospital Israelita Albert Einstein, São Paulo, SP, Brazil.

**Keywords:** Telemedicine, Prehospital care, Length of stay, Transportation of patients, Ambulances, Remote consultation, Patient care team, Communication

## Abstract

**Objective:**

Ambulance transport time is an important metric in prehospital care. Limited studies have explored strategies to decrease on-scene time. We examined the effect of collecting telemedicine-based medical data during ambulance dispatch on the on-scene evaluation time of the prehospital team.

**Methods:**

This randomized, single-center, open-label study included individuals aged >18 years who independently sought hospital emergency services and requested on-site emergency care. Individuals with primary trauma emergencies occurring outside the home, cardiac arrest cases, and situations in which video communication was unfeasible were excluded.

**Results:**

Twenty patients were randomized to receive telemedicine assessment during ambulance dispatch or standard care with physician phone support. Both groups were comparable in age (53.2 ± 26.1 versus 63.4 ± 24.2 years, p=0.380), sex (50% versus 70% female, p=0.360), initial vital signs, and medical history. The main reasons for patients calls were falls from standing height (30%), followed by cardiovascular symptoms (20%), and acute neurological events (15%). Teleconsultation via a mobile application was successfully conducted in all cases. Furthermore, in situ interventions, including venous access, oxygen therapy, orthopedic immobilization, hypotension stabilization, and symptomatic treatment, were similar between the groups. The Telemedicine Group demonstrated a significantly shorter on-scene evaluation time (20.45 ± 6 min) than the Standard Group (36.80 ± 20.4 min, p = 0.019).

**Conclusion:**

Conducting checklist-based anamnesis teleconsultation during ambulance dispatch considerably decreased the on-scene evaluation time of the emergency team. Further research with larger cohorts and different settings is required to better examine telemedicine’s potential in this context.

## In Brief

This pilot study showed that teleconsultation during ambulance dispatch considerably reduced the emergency team’s on-scene evaluation time, underscoring the potential of telemedicine in prehospital care.

## Highlights

■ Teleconsultation reduced on-scene time from 36.8 to 20.6 min (p=0.019).

■ Video-based prearrival anamnesis improved team efficiency.

■ Interventions and clinical profiles were similar across the groups.

■ Study supports broader telemedicine adoption in emergency care.

## Graphical abstract

**Figure:**
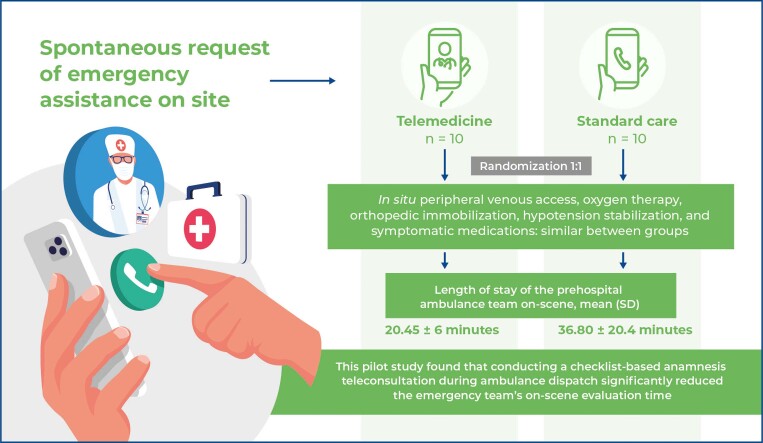

## INTRODUCTION

Prehospital care is a necessary part of the health system, linked to an improved prognosis of several acute diseases, and is most often performed by a multidisciplinary health team during ambulance transport.^1^ The purpose of a prehospital assessment is immediate contact with the patient, rapid diagnostic evaluation, stabilization of initial interventions, and transport to the corresponding emergency facility.^2^

Total transport time is considered an indicator of the fundamental quality of the ambulance transport service, and therefore, of prehospital assessment.^3^ The service can be divided into response time, time spent in the place of precare, and travel time to the emergency unit. The response time (departure from the base until arrival at the place of care) is the most studied and target of most investigations.^4^ Most studies in this context have involved trauma situations, with ample demonstrations of improved prognoses and faster service.^5-7^ However, few controlled and randomized studies exist on prehospital care, primarily in nontraumatic scenarios, with an analysis of the length of stay at the place where care is provided.^8^

Telemedicine (TM), including ambulance transport, is an easy, universal, and low-cost tool for solving several health problems.^9,10^ In the prehospital environment, current evidence reveals that TM support for the ambulance team is related to reduced inappropriate hospital referrals and increased prehospital team availability,^11^improved prognoses for patients with acute myocardial infarction and stroke,^12^ increased local team perception of clinical status and accelerated triage initiation,^10^and changing patterns of transport decisions.^13^

No previous studies have directly addressed the link between TM performed during ambulance travel and the time that the emergency team remains in the place of care. We hypothesized that audio and video teleconsultations with a physician on duty at a TM center could promote clinical data collection and facilitate face-to-face assessments, thereby reducing care time.

## OBJECTIVE

This preliminary study was designed to examine the practicality and preliminary effects of acquiring medical information via teleconsultation during ambulance dispatches on the duration of a prehospital team’s stay at the care site.

## METHODS

### Study design and setting

This unicentric pilot trial used a parallel, randomized, noninferior, open-label design. This study was conducted at *Hospital Israelita Albert Einstein* (HIAE) in São Paulo, (SP), Brazil, which is a private, unreferenced general hospital. The emergency department and TM center are situated at the same institution, with independent routines and professionals. Data were collected and confidentially stored by the TM center separately from the face-to-face prehospital care team.

The local institutional review board approved this study named TeleAmbulance, (CAAE: 44948821.1.0000.0071; # 4.946.628), and all data can be accessed from the institutional digital records.

### Selection of participants

The study population included individuals who spontaneously sought hospital emergency services and requested *in situ* emergency care. Individuals aged >18 years who agreed to participate in the free and informed study were included. Patients with primary trauma emergencies outside the home, patients who experienced cardiac arrest, and patients and companions without a WhatsApp application installed on a cell phone or connection problems that made video communication impossible were excluded.

### Randomization

Patients or their companions contacted the prehospital emergency service at *Hospital Israelita Albert Einstein* via telephone. The local attendant immediately dispatched the ambulance and transferred the call to the emergency doctor on duty at the TM center. The remote physician objectively explained the study to the patient or caregiver on call and requested authorization to participate in the study and randomization. If the patient/caregiver agreed, the patient was randomized using a specific application (Randomizer 1.2, Darshan Institute of Engineering and Technology, Gujarat, India) that provided the random allocation sequence immediately following inclusion in the trial into one of two study groups: teleconsultation or standard.

### Interventions

The TM physician was from a remote emergency team and varied according to duty scale. Eight remote physicians participated in the study. However, the same prehospital staff performed all *in situ* study assessments.

Patients allocated to the TM Group received free audio and video calls via WhatsApp made by the TM physician on duty using an institutional mobile phone. Additionally, the ambulance team was notified and provided feedback to the TM physician when 2 min were left to arrive at the service location, according to the geolocation provided by Google Maps. The remote clinical examination comprised a checklist of current complaints, medical history, current medicine use, and accurate impressions from on-time video assessments. Approximately 2 min before the ambulance arrived, the TM physician completed a video call with the patient, and the clinical data were communicated by telephone to the ambulance staff. Patients in the Standard Group remained exclusively in contact with the TM doctor through audio phone calls and received directions based on their clinical judgment. No information was communicated to the ambulance staff.

The patients or their companions provided informed consent during transport to the hospital or shortly after admission to the emergency room, where clinically possible. Patient participation ended upon arrival at the hospital, with no follow-up. The study ended after the previously estimated sample size was obtained.

### Outcomes

The primary outcome was the length of stay in minutes from the ambulance parking on the scene to the ambulance departure from the patient for the emergency facility. This was counted by an ambulance nurse with a specific wrist stopwatch.

### Analysis

A previous internal observational administrative data analysis showed stability in travel times-approximately 10 min-and time spent at the place of care (approximately 25 min), with low variation. The prehospital team estimated that approximately half of the time spent on-scene was related to anamnesis and the other half to physical and complementary assessments and transportation preparations. Subsequently, it was estimated that patient assessment by teleconsultation was associated with at least a 50% reduction in the performance time of the prehospital team in clinical settings. This study estimated 95% of all necessary clinical data collected by TM assessment during the short time spent in the ambulance based on the typical profile of patients who call the prehospital team at this center. The sample size was obtained to show the noninferiority of the prehospital examination compared with previous TM information, estimating a 50% reduction in the length of hospital stay with a clinically acceptable variation of up to 20%. Enrolling five patients per group provided an approximately 85% chance of detecting a margin of superiority in the primary analysis, with a 5% one-sided significance level. The sample size was increased to 10 patients per group to decrease random errors. A selected patient group was used to assess the feasibility of the proposed intervention, ensuring that it was of sufficient size to evaluate the potential advantages.

IBM-SPSS for Windows version 22.0 was utilized for statistical calculations. The Shapiro-Wilk W test was used to determine the normal distribution of variables. Continuous variables were expressed as means and standard deviations, and categorical variables were presented as counts and percentages. No data were missing. The demographics, first vital signs, medical history, current medicine use, main complaints, and primary outcomes were analyzed using χ^2^, Fisher’s exact, Student’s *t*, and Mann-Whitney U tests. Statistical significance was set at p<0.05.

## RESULTS

### Patients

**Figure 1:**
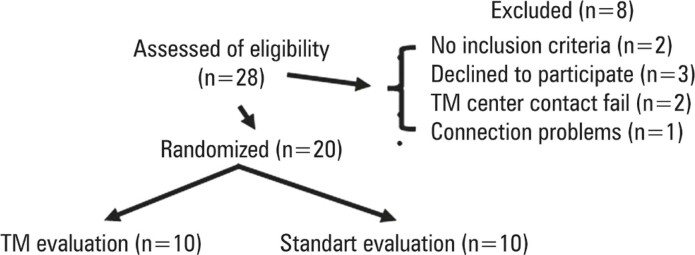
Flow diagram of the study participants

Twenty-eight patients were enrolled from April 16, 2022, to December 10, 2022. Two patients did not meet the inclusion criteria, three withdrew consent, and three showed connection problems. Twenty patients were randomly assigned to receive TM assessments (n=10) during ambulance displacement or standard assessments (n=10) with only physician phone support for the patient or companion (Figure 1).

The two groups were similar regarding age (53.2 ± 26.1 *versus* 63.4 ± 24.2 years, p=0.380), sex (female, 50% *versus* 70%, p=0.360), first vital signs collected *in situ*, and medical history (Table 1). The primary reason for patients calling in both groups was a fall from heights (30%), followed by cardiovascular-related symptoms (20%), and acute neurological manifestations (15%). The main *in situ* interventions included peripheral venous access, oxygen therapy, orthopedic immobilization, hypotension stabilization, and symptomatic medications, all of which were similar in both groups (Table 2). Teleconsultation using a mobile application was feasible in all cases.

**Table 1:** Initial check-listed collected data

Variable	Overall (n=20)	Telemedicine Group (n=10)	Standard Group (n=10)	p value
Age, mean (SD), years	58±25.2	53.2±26.1	63.4±24.2	0.380^‡^
Female sex – n (%)	12 (60)	5 (50)	7 (70)	0.360^£^
First vital signs obtained				
Heart rate, median (IQR), bpm	81 (79-95)	81 (72-96)	84 (80-93)	0.793^#^
Systolic blood pressure, mean (SD), mmHg	124.3±22.72	119±27.06	129±17.58	0.369^‡^
Diastolic blood pressure, mean (SD), mmHg	74.7±15.4	74.7±12.24	75.4±9.92	0.967^‡^
Breath rate, median (IQR), pm	20 (17-22)	22 (20-23)	19 (17-20)	0.020^#^
Oximetry, median (IQR), %	96 (95-98)	96 (95-98)	97 (95-99)	0.367^#^
Capillary blood glucose, mean (SD), mg/dL	112±16.4	106±13.3	118±11.4	0.044^‡^
Main medical history, n (%)				
Neurological medical history	8 (40)	3 (30)	5 (50)	0.368^†^
Cardiac medical history	7 (35)	4 (40)	3 (30)	0.323^†^
Respiratory medical history	5 (25)	3 (30)	2 (20)	0.367^†^
Ongoing antibiotic therapy	3 (15)	2 (20)	1 (10)	0.080^†^

^‡^ Student’s *t* test, ^£^ χ^2^ test, ^#^ Mann-Whitney test, ^†^ Fisher’s exact test.

**Table 2:** Reasons for the call, main in situ interventions, and primary outcomes

Variable	Overall (n=20)	Telemedicine Group (n=10)	Standard Group (n=10)	p value
Main complaints, n (%)				
Fall from their own height	6 (30)	3 (30)	3 (30)	0.999^†^
Acute neurological manifestations	3 (15)	2 (20)	1 (10)	0.088^†^
Painful syndromes	2 (10)	1 (10)	1 (10)	0.999^†^
Cardiac-related symptoms	4 (20)	2 (20)	2 (20)	0.999^†^
Other	5 (25)	3 (30)	2 (20)	0.867^†^
Main *in situ* interventions, n (%)				
Peripheral venous access	14 (70)	7 (70)	7 (70)	0.999^£^
Oxygen therapy	7 (35)	3 (30)	4 (40)	0.765^†^
Orthopedic immobilization	6 (30)	3 (30)	3 (30)	0.999^†^
Hypotension stabilization	5 (25)	3 (30)	2 (20)	0.678^†^
Symptomatic medications	2 (10)	1 (10)	1 (10)	0.999^†^
Primary outcome				
Length of stay of the prehospital ambulance team on-scene, mean (SD), minutes	26±10.36	20.45±6	36.80±20.4	0.019^‡^

^‡^ Student’s *t* test, ^£^ χ^2^ test, ^†^ Fisher’s exact test.

### Primary outcomes

The length of stay of the prehospital ambulance team for on-scene assessment was 20.45 ± 6 min in the TM Group, which was significantly lower than that of 36.80 ± 20.4 min in the Standard Group (p=0.019) (Table 2).

## DISCUSSION

Patient transport centers should provide prehospital care, ensuring less total ambulance time, which is related to improved prognosis.^14^ Emergency medical system administrators must use different strategies to avoid delays in prehospital care to improve the overall efficiency of transportation systems and optimize savings.^15^

Regarding the time spent on-scene, a population-based observational study with 11,585 patients revealed no relationship between age, sex, day of the week, or time of the day with longer times, especially >30 min.^16^ A systematic literature review of eight studies demonstrated greater on-scene prehospital time in rural areas than in urban areas. However, two studies reported no differences in the on-scene time in both environments.^17^

To the best of our knowledge, this is the first randomized study to address a strategy for reducing the on-scene time of prehospital care. We observed a reduction in meaningful on-scene mean time from 36.8 to 20.6 min using a simple strategy: audio-video teleconsultations anticipating critical anamnesis. These detailed data are crucial for the staff team to determine appropriate therapy.^18^ Initial care focused on recognizing clinical deterioration using clinical data and protocol multi-parameter examinations. Recognizing the environment, initial subjective impressions, installing devices, and obtaining objective data require time and can represent barriers to effective communication between patients and staff.^19^ In our study, both groups exhibited similar clinical profiles and *in situ* interventions. A simple anamnesis extracted from TM examinations using a checklist effectively reduced the cognitive load of the ambulance team.^20^The consequence was substantially reduced the on-scene performance time and improved prehospital service quality.

Telemedicine is a low-cost and reproducible approach that connects interfaces in emergency medicine, including ambulance teams. The controlled environment of the TM center makes high-quality service levels more efficient.^21^ Communication within the ambulance team members and effectiveness in safely following TM guidance have been demonstrated.^22^ Herein, the ambulance team effectively assimilated the data collected for the TM Group with mean on-scene interventions similar to those of the Control Group but in considerably less time. No reports of communication issues existed after including the patients in the study. Notably, the study population consisted predominantly of patients with clinical complaints. Patients with cardiac symptoms, acute neurological manifestations, and pain syndromes generally receive a probabilistic diagnosis based on careful anamnesis. These patient profiles increase the relevance of TM evaluation by nonscene *in situ*-related physicians. Interestingly, the effectiveness of the remote examination was method-dependent and not operator-dependent, considering that eight physicians from the TM center participated in the assessments. Notably, no impact existed on the TM center’s on-duty routine, and no dissatisfaction was registered by the remote or face-to-face teams. Despite not being objectively evaluated, the patients and caregivers in this study showed great satisfaction with being examined by a remote doctor via an audio-video connection.

Another relevant discussion concerns prehospital care in nonemergency scenarios. Approximately one-third of patients do not require transportation to a hospital unit.^2^ Inappropriate use of this resource is linked to increased costs and quality loss.^4^ Telemedicine can be used as a filter before ambulance dispatch, redirecting the prehospital team to patients with relevant conditions.^12^All the patients in our study required transportation to a hospital facility. Our study supports the usefulness of TM in more than one aspect of prehospital care.

Remote examination during ambulance dispatch was practical but highly associated with the objective application of a checklist. The checklist approach has considerable evidence of effectiveness in numerous scenarios, including prehospital scenarios.^23^ Additionally, TM plays a crucial role in the correct application of the checklists.^24^

This study has several limitations: the unicentric design, response time, and total transport time were not assessed; the patients were not followed up after arrival at the emergency room; and analyzing the difference in performance among remote physicians was not possible. The sample size was limited given the nature of this pilot study. However, the study population was representative of the standard service profile, and historical transportation times were stable.

## CONCLUSION

In this preliminary study, telemedicine emerged as a practical approach, and conducting anamnesis teleconsultations using a checklist during ambulance dispatch substantially decreased the on-scene duration of the emergency team’s evaluation. Subsequent investigations that incorporate larger participant cohorts and varied environments will be instrumental in further assessing the utility of telemedicine in addressing this matter.
